# Evaluation of an immunochromatography rapid diagnosis kit for detection of chikungunya virus antigen in India, a dengue-endemic country

**DOI:** 10.1186/s12985-018-1000-0

**Published:** 2018-05-11

**Authors:** Jaspreet Jain, Tamaki Okabayashi, Navjot Kaur, Emi Nakayama, Tatsuo Shioda, Rajni Gaind, Takeshi Kurosu, Sujatha Sunil

**Affiliations:** 1 Vector Borne Disease Group, International Centre for Genetic Engineering and Biotechnology, New Delhi, India; 2 Department of Veterinary science, Faculty of Agriculture, University of Miyazaki, Musashimurayama, Japan; 3 Center for Animal Disease Control, University of Miyazaki, Musashimurayama, Japan; 4 Mahidol Osaka Center for Infectious Diseases, Osaka University, Musashimurayama, Japan; 5 Department of Microbiology, Vardhman Mahavir Medical College & Safdarjung Hospital, New Delhi, India; 6 Research Institute of Microbial Diseases, Osaka University, Osaka, Japan; 7 Special Pathogens Laboratory, Department of Virology 1, National Institute of Infectious Diseases, 4-7-1 Gakuen Musashimurayama, Musashimurayama, Japan

**Keywords:** Chikungunya virus, Early diagnosis, Dengue co-infection, Immunochromatography, Mosquito-borne disease

## Abstract

**Background:**

Chikungunya virus (CHIKV) and dengue virus (DENV) are arboviruses that share the same *Aedes* mosquito vector, and there is much overlap in endemic areas. In India, co-infection with both viruses is often reported. Clinical manifestations of Chikungunya fever is often confused with dengue fever because clinical symptoms of both infections are similar. It is, therefore, difficult to differentiate from those of other febrile illnesses, especially dengue fever. We previously developed a CHIKV antigen detection immunochromatography (IC) rapid diagnosis kit [1]. The current study examined the efficacy of previously mentioned IC kit in India, a dengue-endemic country.

**Methods:**

Sera from 104 CHIKV-positive (by qRT-PCR) and/or IgM-positive (ELISA) subjects collected in 2016, were examined. Fifteen samples from individuals with CHIKV-negative/DENV-positive and 4 samples from healthy individuals were also examined. Of the 104 CHIKV-positive sera, 20 were co-infected with DENV.

**Results:**

The sensitivity, specificity and overall agreement of the IC assay were 93.7, 95.5 and 94.3%, respectively, using qRT-PCR as a gold standard. Also, there was a strong, statistically significant positive correlation between the IC kit device score and the CHIKV RNA copy number. The IC kit detected CHIKV antigen even in DENV-co-infected patient sera and did not cross-react with DENV NS1-positive/CHIKV-negative samples.

**Conclusions:**

The results suggest that the IC kit is useful for rapid diagnosis of CHIKV in endemic areas in which both CHIKV and DENV are circulating.

**Electronic supplementary material:**

The online version of this article (10.1186/s12985-018-1000-0) contains supplementary material, which is available to authorized users.

## Background

Chikungunya fever (CF) is caused by chikungunya virus (CHIKV), which belongs to the genus *Alphavirus* and is transmitted to humans by infected mosquitoes. CF is an acute febrile disease with symptoms that include arthralgia, myalgia, headache, vomiting, backache and diffused maculopapular rashes, which are similar to those of dengue fever (DF) [2, 3]. Therefore, it is difficult to diagnose whether an individual is infected with CHIKV or with both CHIKV and dengue virus (DENV). Co-infection of humans and mosquitoes with CHIKV and DENV has been reported in India [4–6]. It is also of concern that several acute febrile diseases such as malaria, influenza, leptospirosis, rickettsiosis, rubella, mycoplasma infections and other febrile diseases are also prevalent in areas in which CF is found; this makes accurate and confident diagnosis of acute febrile diseases more difficult.

Although, several chikungunya rapid diagnostic kits are commercially available, their sensitivity does not always correlate with that of RT-PCR because all of them detect host-derived anti-CHIKV IgM antibodies. Detection of IgM antibodies is less sensitive than detecting antigen as the antibodies are produced later during the course of infection, thereby affecting prompt diagnosis and eventually disease management [7]. Recently, we developed a rapid diagnostic immunochromatography (IC) test kit based on mouse-derived anti-CHIKV monoclonal antibodies that react with a CHIKV East Central South African genotype (ECSA) isolated from patient sera obtained during a CHIKV outbreak in Thailand in 2010 [1, 8]. However, we did not examine the reactivity of these IC kits with serum samples taken from other febrile patients, including those with DF.

The present study was aimed to examine the suitability of the IC kit as a tool for rapid diagnosis of CHIKV in an endemic area, India. For this purpose, we tested the kit during a recent CHIKV outbreak that occurred in New Delhi in 2016.

## Methods

### Virus, cell culture and titrations

CHIKV strain CP10 (ECSA genotype) was propagated in Vero cells maintained in Minimum Essential Medium (Life Technologies, Inc., USA) supplemented with 10% (*v*/v) heat-inactivated Foetal Bovine Serum (Life Technologies). The virus was quantified by quantitative reverse transcription polymerase chain reaction (qRT-PCR) [9], using a laboratory-generated strain (IND/DEL/2010–01) cloned in pGEM-T vector and serially diluted from 100 ng to 1 pg as reference to determine viral copy number of CHIKV viral RNA isolated from patients sera using the formula.

Number of VNA copies = (amount of VNA in nanograms × 6.022 × 10^23^) / (length of VNA amplicon (in basepairs) × 1 × 10^9^ × 330).

The CHIKV strain CP10 was also titrated using standard plaque assay [10].

### Patient recruitment and sample collection

Blood samples were collected from a cohort of suspected dengue and chikungunya patients, with history of fever with joint pains, present within 1 to 15 days of illness and referred to the Department of Microbiology, Vardhman Mahavir Medical College and Safdarjung Hospital, New Delhi, India. In addition, samples from patients suffering from other febrile diseases were collected and used as negative controls for specificity. Also, samples from healthy volunteers were collected as negative controls. The study was jointly funded by the Department of Science and technology (DST), Government of India and Japan Agency for Medical Research and Development (AMED), and all patients and controls signed a consent form approved by the institutional ethical board (IEC/VMMC/SJH/Project/February-2016/574, ICGEB/IEC/2014/01, Version 3). Onset of fever and other clinical features were documented at the time of patient recruitment. Sera were separated from whole blood and stored in − 80 °C.

### Diagnosis of samples and study design

Anti-chikungunya IgM antibodies were detected using Chikungunya-IgM capture ELISA kit (MAC-ELISA; NIV-Pune, India). Also, all samples were subjected to qRT-PCR analysis [9]. Samples that were positive for IgM and/or positive in the qRT-PCR were grouped according to the presence of viral RNA and/or antibodies. The groups are explained in the additional figure file (Additional file 1). Group 1 included all CHIKV samples that tested positive by CHIKV qRT-PCR and/or positive for IgM by ELISA. These samples were then sub-grouped as follows: Group 2, samples positive for CHIKV RNA irrespective of the presence of CHIKV antibodies; Group 3, samples positive for antibodies irrespective of the presence of CHIKV RNA; and Group 4, samples positive for both CHIKV RNA and IgM, DEN IgM and NS1. Samples for other febrile diseases were collected retrospectively after being were diagnosed by following detection methods; Malaria: rapid card test and confirmation by microscopy, Salmonella: Vidal, IgM immunochromatography test and culture, HIV: 4th generation ELISA, Leptospirosis: rapid IC test and Influenza: PCR. These samples were used to detect cross-reactivity of the IC kits.

### Testing the IC kit

Thirty microliters of serum or ten-fold serially diluted CHIKV culture supernatant was placed in a tube and mixed with 30 μl of IC kit extraction buffer (supplied by ARKRAY, Inc. Kyoto, Japan). The IC kit was inserted into the tube and developed chromatographically. After 15 min, two independent researchers examined the control and test lines visually and in a blinded manner. Then, an IC Reader C10066–10 (Hamamatsu Photonics K.K., Japan) was used to measure the actual intensity of the test lines.

### Statistical analysis

The correlation between the test device score and the CHIKV RNA copy number measured by qRT-PCR was analysed and the significance of the correlations was estimated using the Pearson correction; *P* < 0.05 was considered to be significant. All data were analysed using statistical software R 3.3.3 (The R Foundation, https://www.r-project.org/).

## Results

### Testing the IC kit using clinical samples

The detection limit of the IC kit was determined using serially diluted CHIKV recovered from cell culture supernatants; the limit was approximately 10^4^ PFU/ml. Additional figure file explains limit of detection of the immunochromatography rapid diagnosis kit for chikungunya virus antigen in more detail (Additional file 2). From sera collected for this study, total 104 (Group 1) were diagnosed as CF by either qRT-PCR or IgM. These 104 sera (Group 1) were further categorized into 3 groups, based on different combinations of test: 1) Group 2 (*n* = 79), CHIKV positive by qRT-PCR, 2) Group 3 (*n* = 50), CHIKV positive by IgM, 3) Group 4 (*n* = 25), CHIKV positive by both qRT-PCR and IgM. Efficacy of IC kit was assessed compared to qRT-PCR result serving as the gold standard. The sensitivity, specificity and overall agreement (OAA) of the IC kit were evaluated to each group 1–4, respectively, and those for Group 1 were 72.1, 94.7 and 75.6%, respectively (Table 1). All samples from healthy volunteers were negative by IC kit (*n* = 4). The IC kit targets viral antigen, CHIKV E protein. To compare efficacy of IC kit to other antigen detection method, CHIKV positive sera by qRT-PCR (Group 2) were extracted from Group 1. For Group 2, the sensitivity, specificity and OAA of the IC kit for CHIKV positive sera by qRT-PCR (irrespective of the presence of anti-CHIKV IgM antibodies) were 93.7, 95.5 and 94.3%, respectively. For comparison, efficacy of the IC kit was calculated against CHIKV positive sera by IgM (Group 3). The sensitivity, specificity and OAA of the IC kit for CHIKV positive sera by IgM (irrespective of the presence of CHIKV RNA) were 46.0, 27.4 and 35.0%, respectively. Low sensitivity of the IC kit to Group 3 may be due to the presence anti-CHIKV IgM antibodies. To test this, we assessed the sensitivity using Group 4 sera (CHIKV positive by both qRT-PCR and IgM). There was no significant reduction in the sensitivity of the IC kit for sera in Group 4 (88.0%). The existence of IgM did not affect the sensitivity of IC kit if samples contained qRT-PCR detectable viral antigen.

**Table 1:** Sensitivity, specificity and over all agreement with real time RT-PCR of immunochromatography kit for chikungunya virus antigen using clinical serum samples in various clinical categories

	Criteria of CHIKV positive	Sensitivity (%)	Specificity (%)	OAA^*1^ (%)
Group 1^*2^	qRT-PCR or IgM qRT-PCR and/or IgM	72.1 (75/104)	94.7 (18/19)	75.6
Group 2	qRT-PCR	93.7 (74/79)	95.5 (42/44)	94.3
Group 3	IgM	46.0 (23/50)	27.4 (20/73)	35.0
Group 4	qRT-PCR and IgM	88.0 (22/25)	44.9 (44/98)	53.7

The sensitivity, specificity and overall agreement of IC kit were calculated using qRT-PCR as a gold standard. ^*1^ OAA: Overall agreement with real time RT-PCR. * [2] Group 1 includes three groups, qRT-PCR and IgM positive group, either qRT-PCR positive or IgM positive

### Comparison of IC kit performance with that of qRT-PCR and IgM ELISA using confirmed CF samples

**Fig. 1:**
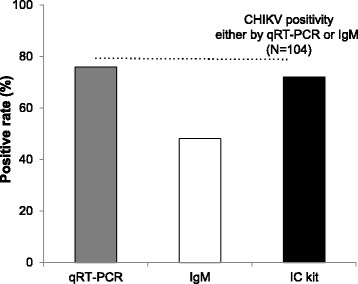
Comparison of qRT-PCR, IgM ELISA and Immunochromatography test (IC) kit results when used to test confirmed CHIKV samples (*n* = 104). Serum samples that gave a positive result in the qRT-PCR and/or IgM ELISA assays were considered CHIKV-positive (dotted line, 100%)

Patients visit hospitals at different times after onset of fever. Therefore, to examine the clinical utility of the IC, we compared its results with those from qRT-PCR and IgM ELISA using samples from Group 1. The positive detection rates for the qRT-PCR, IgM ELISA and IC kit were 76.0, 48.1 and 72.1%, respectively (Fig. 1). Therefore, the detection rate of the IC kit was similar to that of qRT-PCR, and much higher than that of the IgM ELISA.

### Correlation between the test device scores and the results from the IC kit

**Fig. 2:**
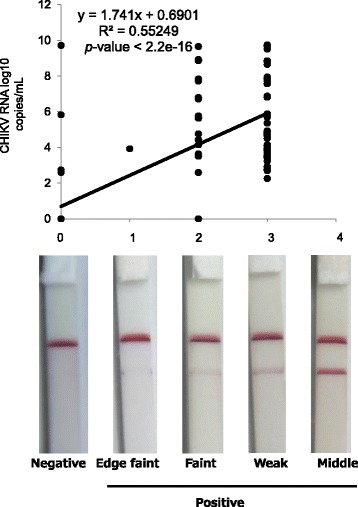
Correlation between the test device score and the results of the Immunochromatography test (IC) kit. There was a significant positive correlation between the two results. Therefore, the IC kit results correlated with the CHIKV RNA copy number (correlation coefficient (*r*), 0.55249; *p* < 2.2e-16)

The relationship between the IC kit results (depicted as scores) and the copy number of CHIKV RNA (from qRT-PCR) was examined (Fig. 2). There was a positive correlation between the two (*p* < 2.2e-16, Fig. 2). The score from the IC kit reflected the viral copy number in clinical samples.

### Detection of CHIKV antigen, RNA and IgM antibodies with respect to the time of fever onset

**Fig. 3:**
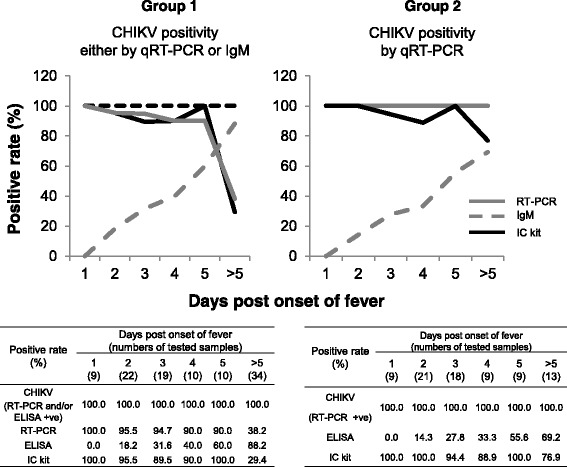
Comparison of results from qRT-PCR, IgM ELISA and the Immunochromatography test (IC) kit when used to test confirmed CHIKV samples (n = 104). Serum samples that gave a positive result by qRT-PCR and/or IgM ELISA were considered CHIKV-positive (Group 1; dotted line = 100%). Panels show the percentage of samples positive for CHIKV antigen, RNA and IgM antibodies (Groups 1 and 2) with respect to the time of fever onset

The percentage of positive samples detected by the IC kit, qRT-PCR and IgM ELISA at different times after fever onset is shown in Fig. 3. The data from the IC kit were in agreement with those from qRT-PCR of confirmed CF samples. When tested against Group 1 samples, the positive detection rates of the IC kit and of qRT-PCR fell at 6 days post-fever onset: qRT-PCR, 38.2%; IgM, 88.2%; IC kit, 29.4%. However, when tested against Group 2 samples, the positive detection rate of the IC kit remained high, even 6 days after fever onset (76.9%). Taken together, the data suggest that the IC kit and qRT-PCR detect positive samples up until 5 days post-fever onset.

### Specificity test using samples from patients with dengue

To examine the specificity of the IC kit, we used it to test sera from patients diagnosed as dengue-positive (DENV NS1 positive). Sera from CHIKV/DENV-co-infected patients were assigned to one of three groups: D1) CHIKV positive by qRT-PCR an IgM; D2) CHIKV positive by qRT-PCR, not IgM; and D3) CHIKV positive by IgM, not qRT-PCR (Table 2). The IC kit detected CHIKV antigen, even in DENV-co-infected sera containing CHIKV RNA, in the presence or absence of CHIKV IgM (Groups D1 and D2); this was not the case for the CHIKV RNA-negative samples (Group D3). The IC kit detected only one false-positive serum sample in the DENV NS1positive but CHIKV negative (Group D4). Furthermore, we tested the cross-reactivity of the IC kit using 44 sera derived from patients with other fibril diseases (e.g., malaria, typhoid, hepatitis B or C, and Salmonella), which are also endemic to India. The IC kit did not react with any of these samples (data not shown).

**Table 2:** Use of the immunochromatography kit to detect chikungunya virus antigen in sera co-infected with dengue virus

	Group	Criteria	Sample Numbers	IC kit Positive Numbers	(%)
CHIKV+DENV co-infection	Group D1	CHIKVq RT-PCR + ve /CHIKV IgM + ve	3	3	100
	Group D2	CHIKVq RT-PCR + ve /CHIKV IgM-ve	6	6	100
	Group D3	CHIKV qRT-PCR-ve /CHIKV IgM + ve	11	0	0
DENV	Group D4	CHIKVq RT-PCR-ve /CHIKV IgM-ve	15	1	6.6

## Discussion

Since its first report in Delhi in 2009, a total of 272,384 confirmed CF patients have been reported [11]. Apart from these CF outbreaks that occur from time to time, the additional burden of co-infections with DENV makes patient management a huge concern [12] The current approach of CF diagnosis is either by detecting the viral RNA using quantitative PCR or by detecting the antibodies against the virus using ELISA. Both the above-mentioned methods have their own caveats making early diagnosis of CF cumbersome and/or ineffective, especially during outbreaks involving large number of individuals. Through the present report we provide evidence of the antigen-based IC kit as a valuable tool for early diagnosis of CF in Indian patients that can replace the current approach of qRT-PCR detection. While the kit did not cross-react with serum samples from patients infected with other febrile diseases including DENV, we show that this kit is effective in detecting CHIKV in patients co-infected with DENV. Whereas the detection rate of the IC kit was similar to that of qRT-PCR, the IC kit provides a CF diagnosis in between than 15–30 min making it much faster than qRT-PCR (or ELISA) and making this kit comparable to other early diagnostic tools available for other febrile fevers such as dengue [13]. Owing to its rapid detection time, this kit may also be used as a point-of-care kit especially in outbreak situations.

The detection rate of the IC kit fell at 6 days post-fever onset (Fig. 3). It is probably because the IC kit detects viral envelop protein which generally drops after 4–5 days post-infection. At the same time, the production of IgM increases as a host response that could probably hamper antigen detection. As most patients visit the clinic early while they are still in the pyrexic phase of the disease, we believe that the detection rates of the IC kit as recorded in this study are satisfactory to be used during the outbreaks in the chikungunya endemic countries.

The MAb used in the IC kit was produced by immunization of the ECSA strain isolated in Thailand [1]. Mutation analysis of this strain revealed some substitutions located within the epitope region of E1 gene for anti-CHIKV antibody used in the IC kit. Results from the current study reveal that these changes do not impact the kit performance. Analysis of the E1 gene from the Indian isolates reveal that these variations were not present in these isolates thereby providing confidence to the utility of this kit in Indian patients [14]. Our results suggest that the IC kit could be used for early CF diagnosis not only in Southeast Asian areas but also Indian Ocean areas as well, where CHIKV ECSA is endemic.

## Conclusions

The IC kit tested herein detected ECSA genotypes of CHIKV present in India during the early phase of the disease suggesting that this kit could be used during outbreak situations in endemic regions. The IC kit did not cross-react with sera from patients infected with DENV alone and it detected CHIKV antigen in co-infected cases. Thus, the IC kit may be useful in areas in where the CHIKV ECSA genotype is endemic and have high probability to occur as co-infections with dengue.

## Additional files

Additional file 1: Figure file explaining the flow chart of sample collection and testing. The flow chart shows the number of CF-or dengue-suspected samples (*n* = 119) and sera from healthy donors (*n* = 4). CF-or dengue-suspected samples were diagnosed by NS1 ELISA (dengue) or IgM ELISA and qRT-PCR (CHIKV). One hundred and four samples were diagnosed as CHIKV-positive (Group 1). Among these, 79 were diagnosed as positive by qRT-PCR (Group 2) and 50 by IgM ELISA (Group 3). Twenty-five samples were diagnosed as positive by both qRT-PCR and IgM ELISA (Group 4). The test line generated by the IC kit was inspected visually by two researchers (blinded to each other). (PDF 410 kb)

Additional file 2: Figure file depicting limit of detection of the immunochromatography rapid diagnosis kit for chikungunya virus antigen. Virus recovered from cell culture was used to test the IC kit. The intensity of the test lines (upper panel) was measured in an IC Reader C10066–10 (lower panel). (PDF 346 kb)
