# Supplementary material for: Evaluation of an immunochromatography rapid diagnosis kit for detection of chikungunya virus antigen in India, a dengue-endemic country
Source: Virol J. 2018 May 11;15:84. doi: 10.1186/s12985-018-1000-0 (PMC5948817; doi:10.1186/s12985-018-1000-0)
Supplement: Supplementary file 1 — Figure file explaining the flow chart of sample collection and testing. The flow chart shows the number of CF-or dengue-suspected samples (n = 119) and sera from healthy donors (n = 4). CF-or dengue-suspected samples were diagnosed by NS1 ELISA (dengue) or IgM ELISA and qRT-PCR (CHIKV). One hundred and four samples were diagnosed as CHIKV-positive (Group 1). Among these, 79 were diagnosed as positive by qRT-PCR (Group 2) and 50 by IgM ELISA (Group 3). Twenty-five samples were diagnosed as positive by both qRT-PCR and IgM ELISA (Group 4). The test line generated by the IC kit was inspected visually by two researchers (blinded to each other). (PDF 410 kb) [file 12985_2018_1000_MOESM1_ESM.pdf]

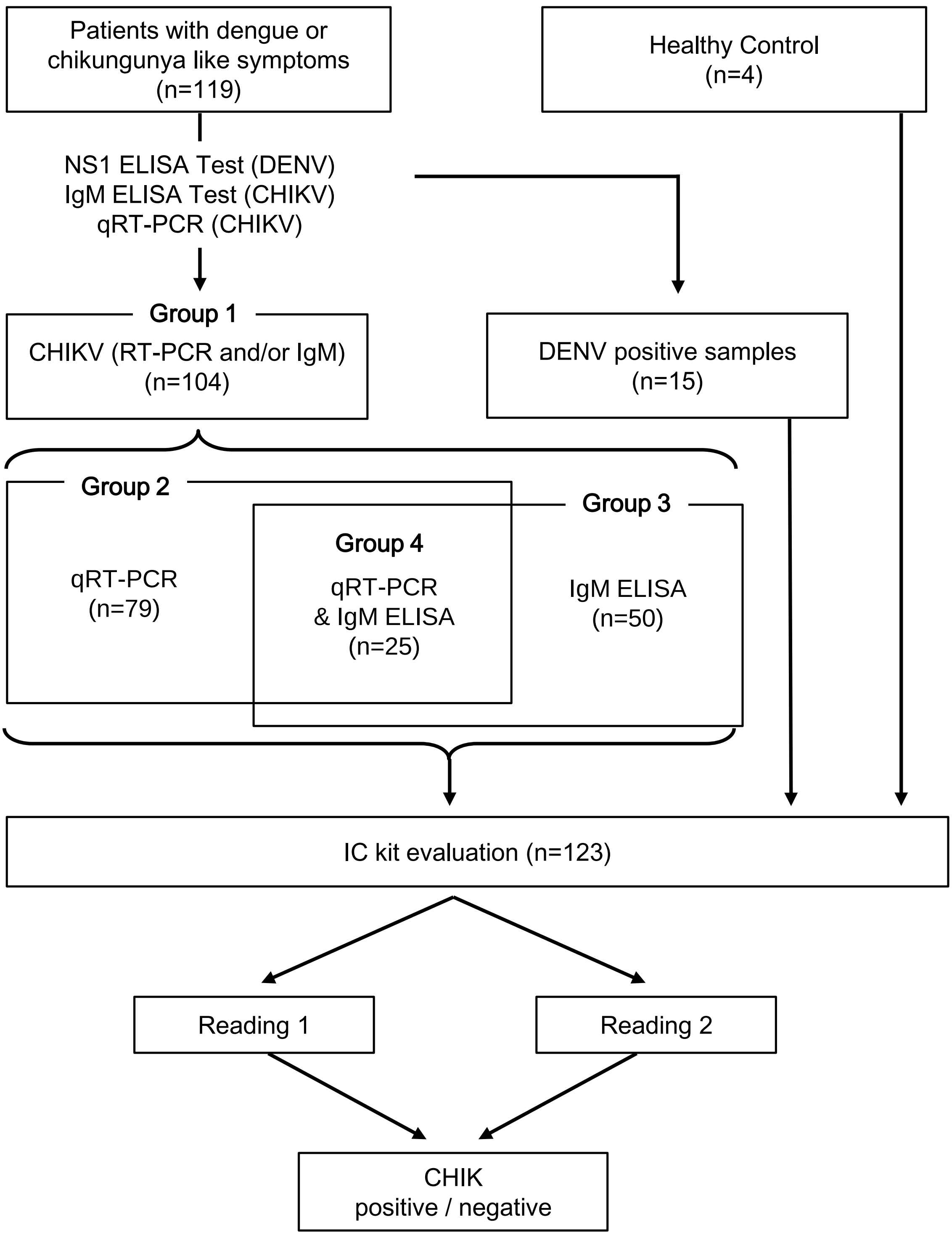

Supplementary Figure 1. Flow chart of sample collection and testing. The flow chart shows the number of CF- or dengue-suspected samples (n=119) and sera from healthy donors (n=4). CF- or dengue-suspected samples were diagnosed by NS1 ELISA (dengue) or IgM ELISA and qRT-PCR (CHIKV). One hundred and four samples were diagnosed as CHIKV-positive (Group 1). Among these, 79 were diagnosed as positive by qRT-PCR (Group 2) and 50 by IgM ELISA (Group 3). Twenty-five samples were diagnosed as positive by both qRT-PCR and IgM ELISA (Group 4). The test line generated by the IC kit was inspected visually by two researchers (blinded to each other). Author: Please include the group designations here.
