# Supplementary material for: Evaluation of an immunochromatography rapid diagnosis kit for detection of chikungunya virus antigen in India, a dengue-endemic country
Source: Virol J. 2018 May 11;15:84. doi: 10.1186/s12985-018-1000-0 (PMC5948817; doi:10.1186/s12985-018-1000-0)
Supplement: Supplementary file 2 — Figure file depicting limit of detection of the immunochromatography rapid diagnosis kit for chikungunya virus antigen. Virus recovered from cell culture was used to test the IC kit. The intensity of the test lines (upper panel) was measured in an IC Reader C10066–10 (lower panel). (PDF 346 kb) [file 12985_2018_1000_MOESM2_ESM.pdf]

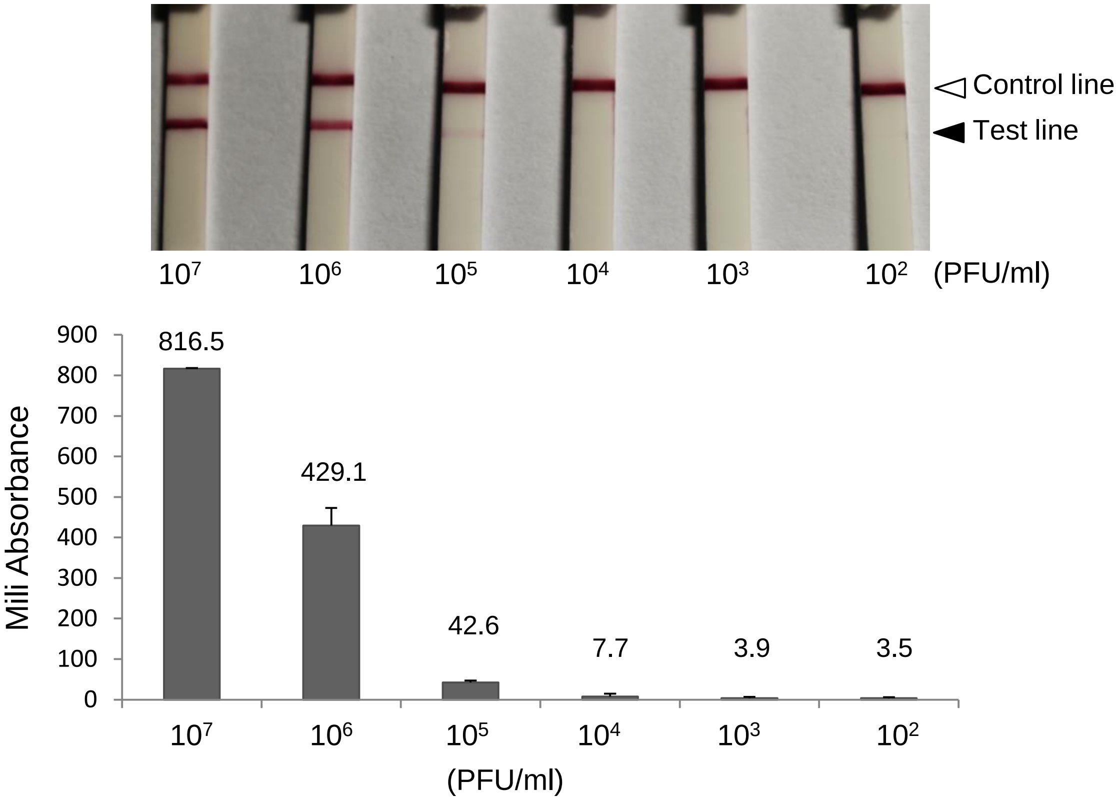

Supplementary Figure 2. Limit of detection of the immunochromatography rapid diagnosis kit for chikungunya virus antigen. Virus recovered from cell culture was used to test the IC kit. The intensity of the test lines (upper panel) was measured in an IC Reader C10066-10 (lower panel).
